# The Impact of DeBakey Forceps on Cardiac Surgery: Revolutionizing the Surgical Landscape

**DOI:** 10.7759/cureus.63121

**Published:** 2024-06-25

**Authors:** Shahbaz Salehi

**Affiliations:** 1 Infectious Diseases, Foothill Regional Medical Center, Irvine , USA

**Keywords:** tissue, tissue viability, open heart surgery, clamp and retract, medical instruments, operative instruments, debakey, forceps, adult cardiac surgery, instruments

## Abstract

The evolution of cardiac surgery has been marked by significant advancements in surgical techniques and tools, leading to improved patient outcomes and safety. A pivotal development in this journey has been the creation of DeBakey forceps, an instrument designed by Dr. Michael DeBakey, a prominent cardiac surgeon and medical innovator. This review explores the impact of a simple, yet effective surgical force invented by Dr. DeBakey, which is not only a cornerstone of cardiac surgery but also finds applications in various other specialties, piquing curiosity about its versatility and unique design that has revolutionized the field of surgery.

## Introduction and background

Michael Ellis DeBakey, an iconic American surgeon, educator, and medical statesman, left an indelible mark on cardiovascular surgery and significantly influenced medical education and healthcare policy over a career spanning 75 years. He pioneered numerous lifesaving surgical techniques, including aneurysm repair, coronary bypass, and endarterectomy, and performed some of the first heart transplants. His innovations also extended to medical technology, with his roller pump becoming a key component of heart-lung machines, and he contributed to the development of artificial hearts and ventricular assist pumps. Beyond his surgical achievements, DeBakey played a central role in establishing the Baylor College of Medicine in Houston as a premier medical institution, where he trained generations of top surgeons worldwide, thereby impacting the global medical community [1,2,3].

**Figure 1:**
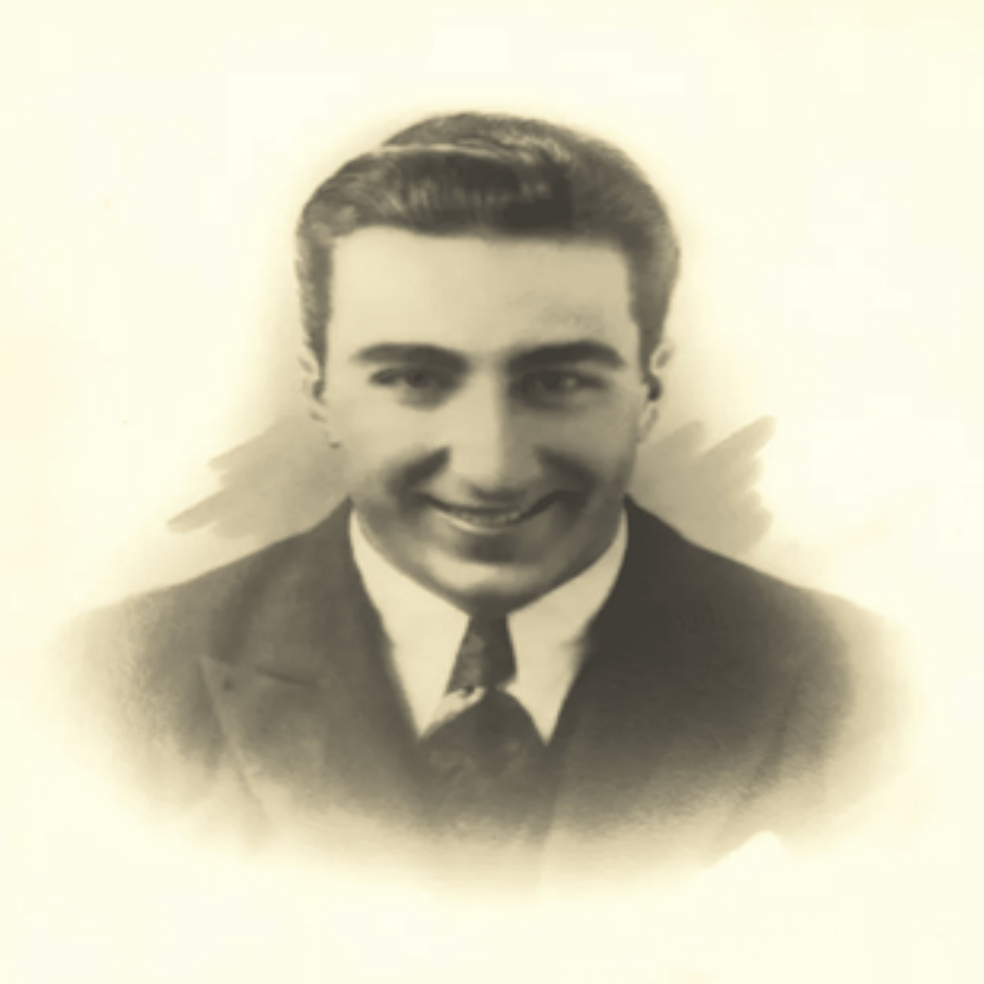
Michael DeBakey, age 20 years, 1928. Source: [1]. Courtesy:  Katrin DeBakey (family member).

Born in Lake Charles, Louisiana, in 1908, DeBakey was the eldest child of Lebanese immigrants. His early years were marked by a profound passion for learning and exploration, instilled by his parents, who fostered his intellectual curiosity and compassion. His journey into medicine began during his boyhood, fueled by his experiences working in his father's drugstore, where he had the privilege of interacting with visiting physicians. DeBakey's academic journey took him to Tulane University, where he earned his MD in 1932, and later to Europe, where he studied under renowned surgeons. He eventually joined the Tulane faculty, where he met his first wife, Diana Cooper, and started his family (Figure 1).

During World War II, DeBakey's expertise contributed to developing medical strategies that improved surgical care for wounded troops. His work with the Surgical Consultants Division of the Army Surgeon General's Office laid the foundation for the Mobile Auxiliary Surgical Hospital (MASH) units used in the Korean and Vietnam conflicts. After the war, DeBakey continued his public service, contributing to establishing the Veterans Administration hospital system and playing a pivotal role in improving governmental healthcare programs through his work with the Hoover Commission. His advisory roles extended to the National Institutes of Health and several U.S. presidents, including Lyndon B. Johnson [1,2].

**Figure 2:**
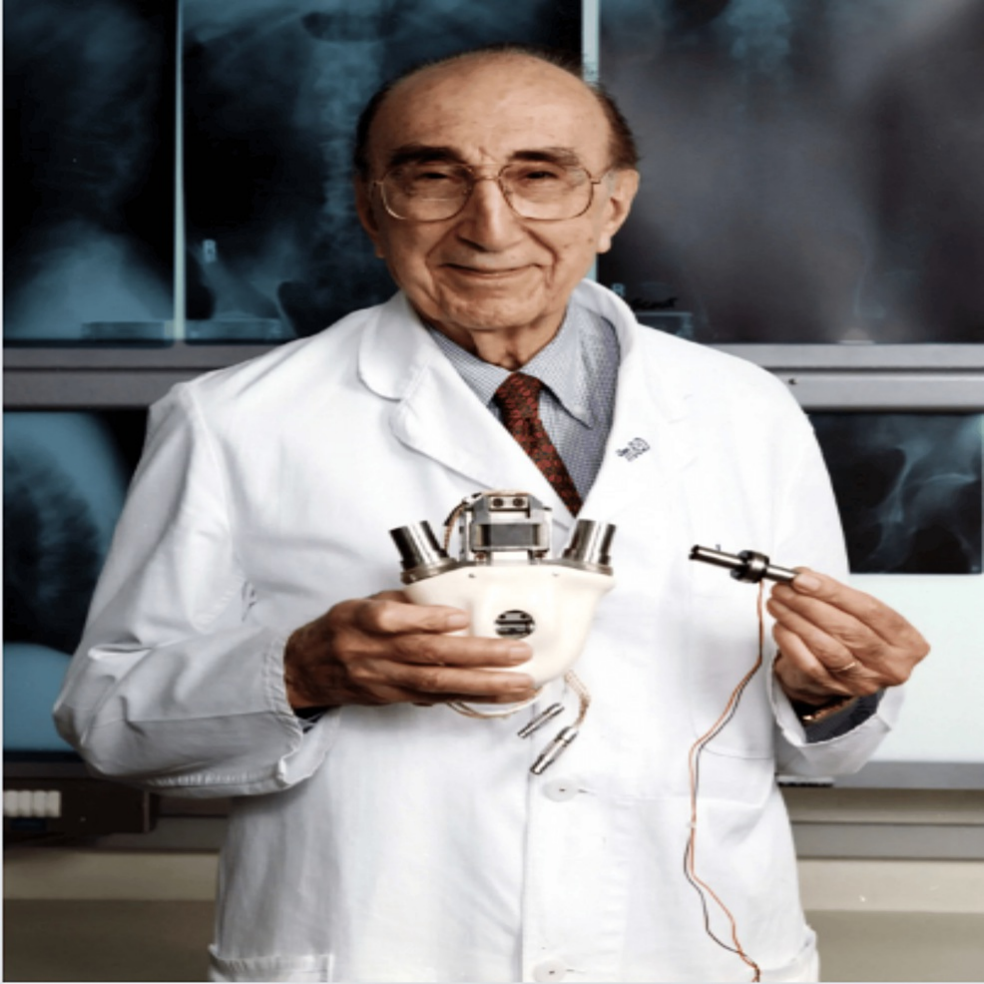
Dr. DeBakey holding an artificial heart. Source: [8]. Courtesy: Academy of Achievement (https://achievement.org/achiever/michael-e-debakey-m-d/).

DeBakey's groundbreaking work in cardiovascular surgery brought international recognition and prestige to the Baylor College of Medicine. His successful repair of abdominal aortic aneurysms, pioneering use of Dacron grafts, and development of open-heart surgery techniques were landmark achievements. Despite a rigorous schedule, DeBakey also focused on research and developing cardiac assist devices, including the DeBakey VAD, a miniaturized axial-flow ventricular assist device. Throughout his career, DeBakey authored over 1,600 publications and received numerous accolades, including the Presidential Medal of Freedom and the Congressional Gold Medal. He continued performing surgery until 90 and died in 2008, leaving behind a legacy of innovation and excellence in cardiovascular surgery and beyond. Among his countless innovations, one often overlooked yet profoundly impactful contribution to surgical practice is the creation of DeBakey forceps. This modification in design not only safeguards the integrity and vitality of delicate tissues but also prioritizes ergonomic comfort for the surgeon, ensuring a natural feel during use. Such considerations are especially crucial in procedures involving blood vessels and soft tissue. Despite their simplicity, DeBakey forceps remain a cornerstone in modern surgical techniques (Figure 2) [1,2].

## Review

Description and design of DeBakey forceps

The traditional forceps available during DeBakey's early career were often designed with serrated edges, which, while useful for gripping, could cause unnecessary trauma to delicate tissues and blood vessels. DeBakey forceps are designed with specific characteristics that are ideal for fragile and precise tasks in cardiac surgery. These forceps are typically long and slender and have a nonserrated surface, which allows for gentle handling of delicate tissues and vessels. The lack of serrations minimizes the risk of crushing or damaging blood vessels during surgical procedures, a critical feature in cardiac surgery, where precision is paramount. These forceps excel in securely gripping tissue with strength while minimizing trauma. Their multiple fine serrations function like a traditional *bed of nails*, distributing the grasping pressure across numerous points to prevent tissue penetration [3,4,5,6,7].

How DeBakey forceps revolutionized cardiac surgery

The introduction of DeBakey forceps marked a significant turning point in cardiac surgery for several reasons. One of the primary benefits of DeBakey forceps is their ability to manipulate tissues and vessels without causing considerable damage. The nonserrated design allows surgeons to grasp and move delicate structures without crushing or tearing them. This is especially crucial in cardiac surgery, where damage to blood vessels can lead to complications such as bleeding, thrombosis, or tissue necrosis. Additionally, DeBakey forceps provide enhanced precision and control, which is essential for the meticulous attention to detail and precise movements required in cardiac surgery. The slender design allows surgeons to reach deep into the surgical field, while the fine tips enable them to manipulate small structures accurately. These forceps also improve surgical outcomes by reducing tissue damage and enhancing precision, leading to fewer complications, reduced recovery times, and better overall results for cardiac surgery patients. This has contributed to increased success rates in cardiac procedures and the advancement of the field. Furthermore, DeBakey forceps are versatile instruments used in various surgical contexts beyond cardiac surgery, including vascular surgery, neurosurgery, and general surgery, making them a staple in operating rooms worldwide [3-15].

The design

**Figure 3:**
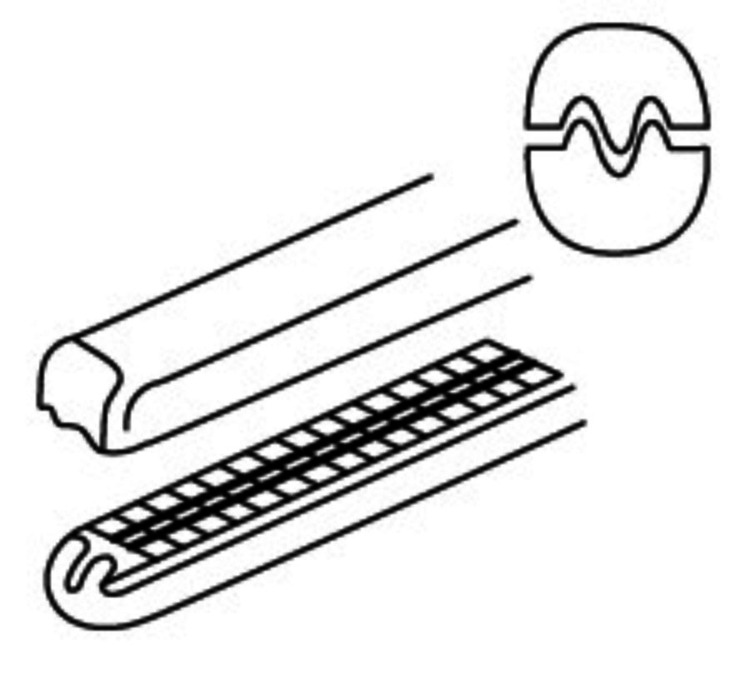
Atraumatic teeth grip tissue, vessels, and organs without damage. Credit: Permission obtained from World Precision Instruments (https://www.wpiinc.com/var-501239-debakey-tissue-forceps-15cm.html).

The primary advantage of these forceps lies in incorporating small serrations along the forcep's jaws, a design feature meticulously engineered to mitigate the risk of tissue injury during delicate procedures involving vascular specimens. These finely crafted serrations ensure a secure grip and enable precise handling, which is crucial for maintaining tissue integrity (Figure 3) [15].

Moreover, the forceps have large metal grooves along the handle, strategically positioned to enhance the surgeon's control and maneuverability. This feature empowers the surgical team to execute intricate movements confidently and accurately, even in the most challenging surgical scenarios [6].

Additionally, the flat, semicircular end of the forceps serves a versatile purpose, allowing for gentle yet effective blunt manipulation of tissue when necessary. Whether repositioning delicate structures or navigating intricate anatomical landscapes, this multifunctional component enhances the versatility and utility of the forceps in various surgical contexts.

In essence, the meticulous design of these forceps prioritizes patient safety through minimized tissue trauma and facilitates surgeon proficiency and precision, ultimately enhancing the overall quality of surgical outcomes.

**Figure 4:**
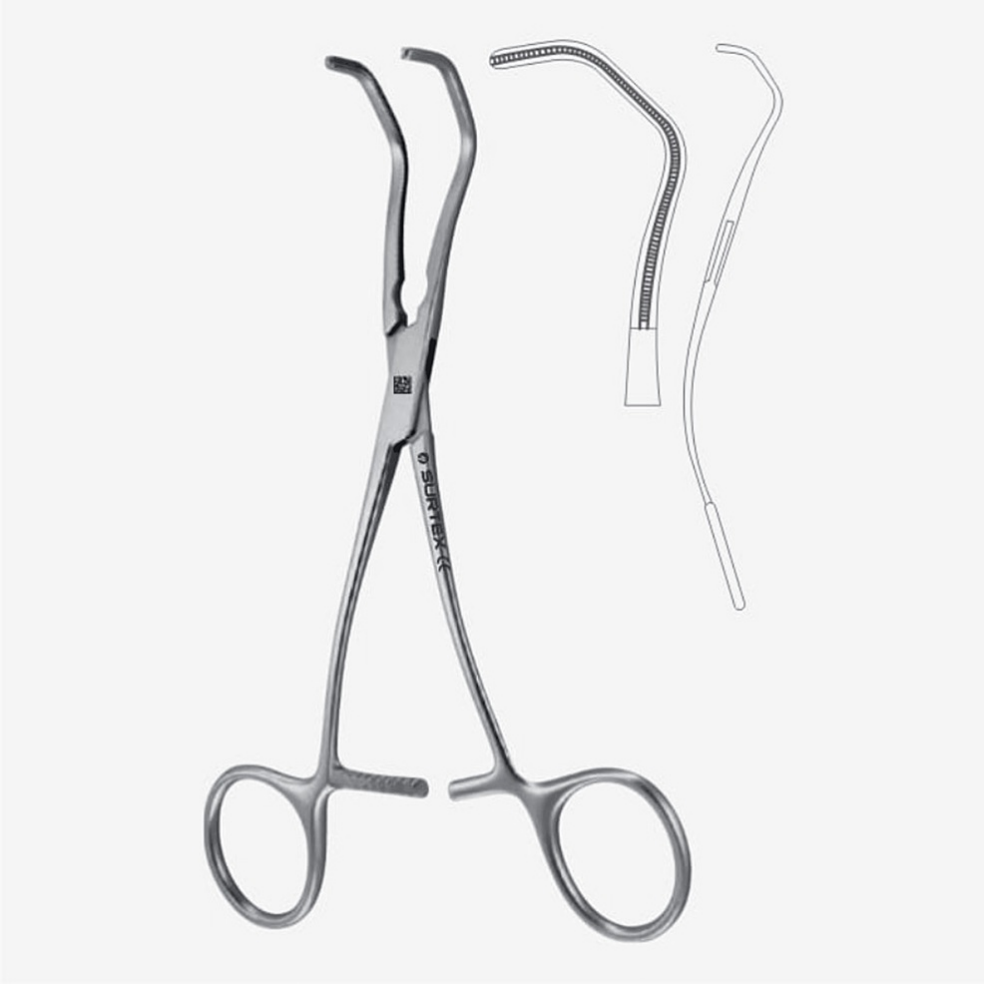
DeBakey atruma anastomosis clamp. Image credit: Permission obtained from Surtex Instruments (https://surtex-instruments.com/product/debakey-atrauma-anastomosis-clamps/).

**Figure 5:**
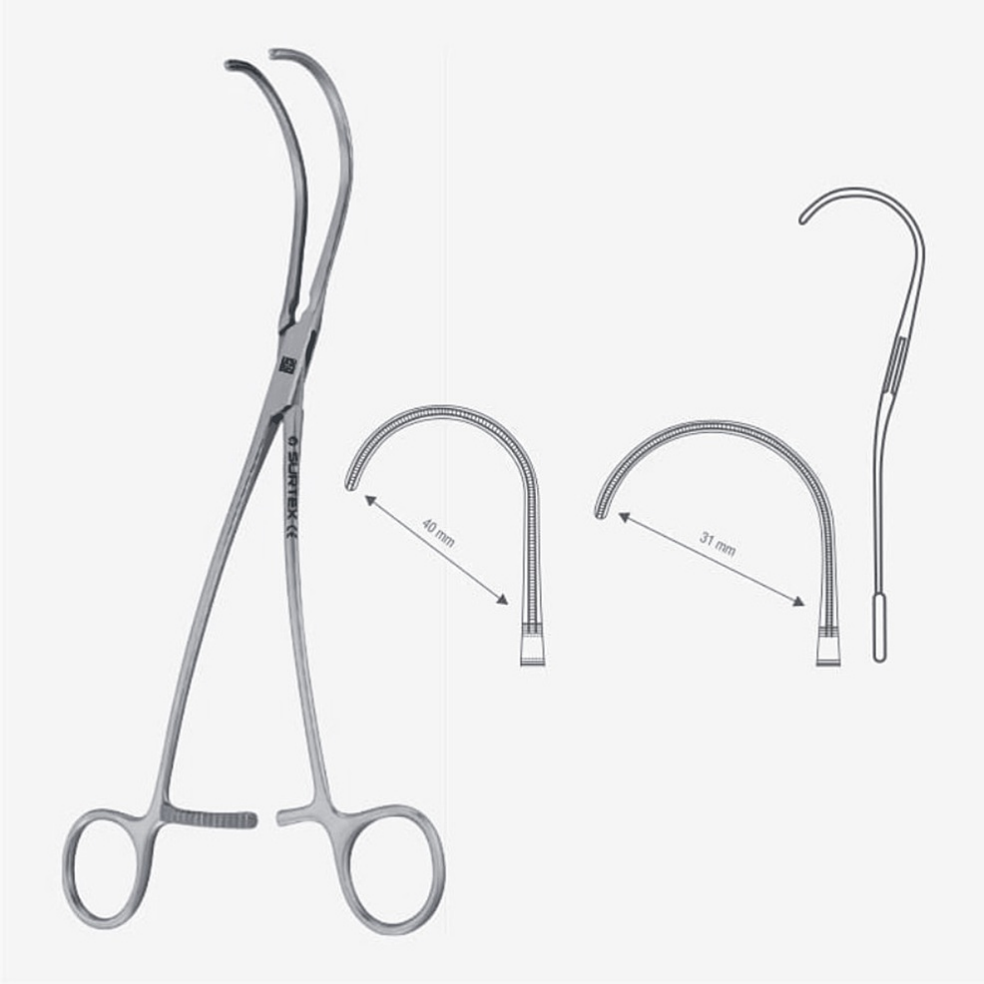
DeBakey atraumatic multipurpose vascular clamp. Image credit: Permission obtained from Surtex Instruments (https://surtex-instruments.com/product/debakey-atraumatic-multipurpose-vascular-clamps/).

This distinctive design has not only revolutionized forceps but has also been successfully applied to surgical clamps. The innovative features, such as the small serrations on the clamp jaws, are adeptly engineered to minimize tissue trauma during clamping procedures, particularly in delicate surgical interventions involving intricate vascular or soft tissue structures (Figures 4-5).

Integrating these finely tuned serrations ensures a secure yet gentle grip on tissues, safeguarding their integrity throughout the surgical process. Moreover, like the forceps, the clamps boast strategically placed metal grooves on their handles, providing surgeons with heightened control and precision during critical maneuvers [6].

Furthermore, this design's versatility extends to the clamps' flat, semicircular end, allowing nuanced tissue manipulation without compromising delicate structures. Whether securing vessels or temporarily occluding blood flow, these clamps deliver unparalleled performance and reliability in diverse surgical settings.

In essence, applying this unique design to surgical clamps underscores its adaptability across various surgical instruments and exemplifies its enduring impact on advancing surgical techniques and patient care.

Ergonomic aspect of the instrument

The ergonomics of surgical instruments play a pivotal role in enhancing the surgeon's performance and patient outcomes. Ergonomics focuses on optimizing the interaction between individuals and their environment, aiming to minimize discomfort, fatigue, and the risk of injury while maximizing efficiency and precision [15].

Ergonomic design becomes paramount in the surgical setting, where intricate procedures demand unwavering concentration and dexterity. Ergonomic instruments are tailored to fit comfortably in the surgeon's hand, promoting natural movements and reducing strain during prolonged surgeries. This enhances the surgeon's comfort and minimizes the likelihood of hand fatigue and musculoskeletal injuries, allowing for sustained focus and steady performance throughout the procedure.

**Figure 6:**
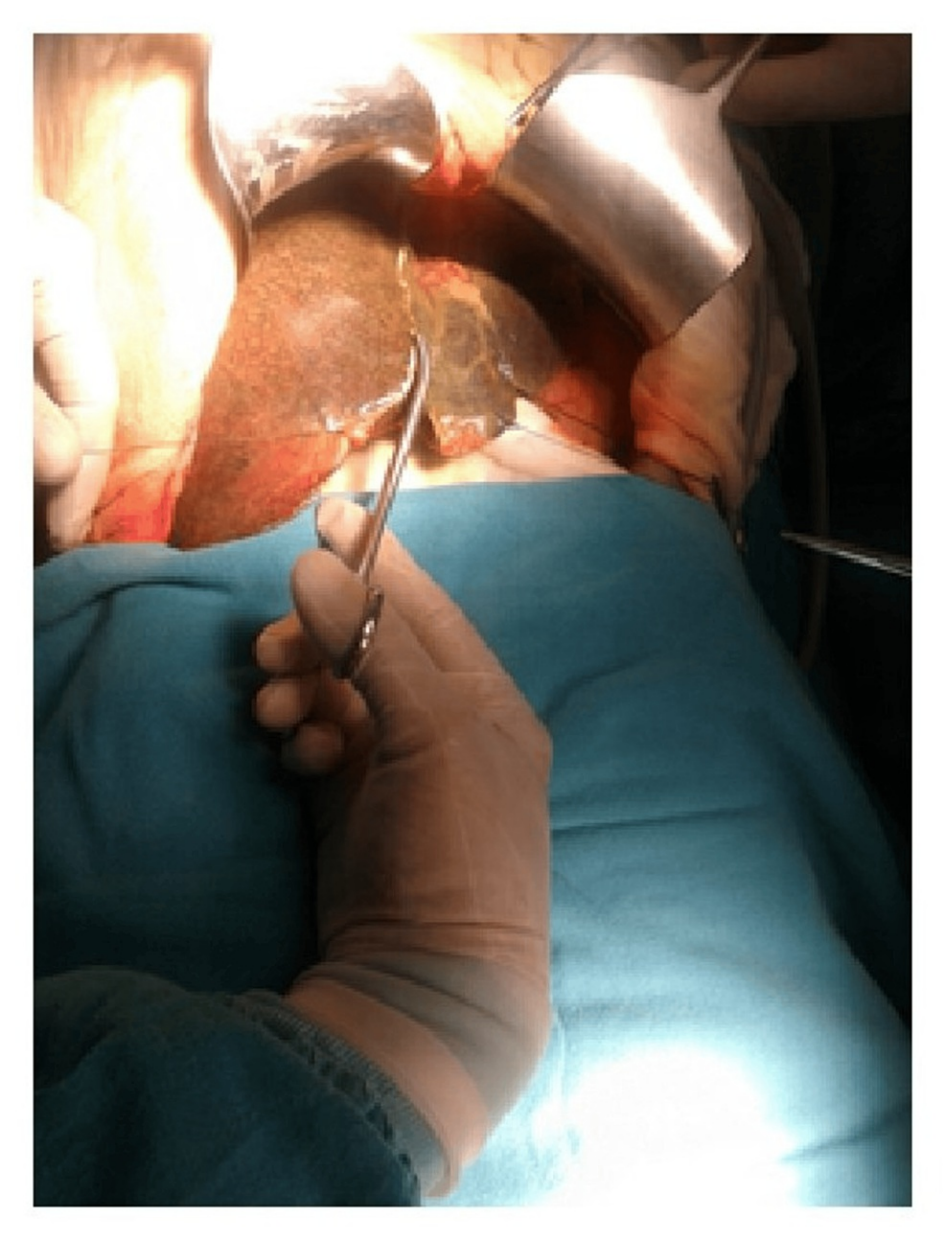
Abnormal posture of the wrist while using the Kelly clamp. Image credit: [6].

**Figure 7:**
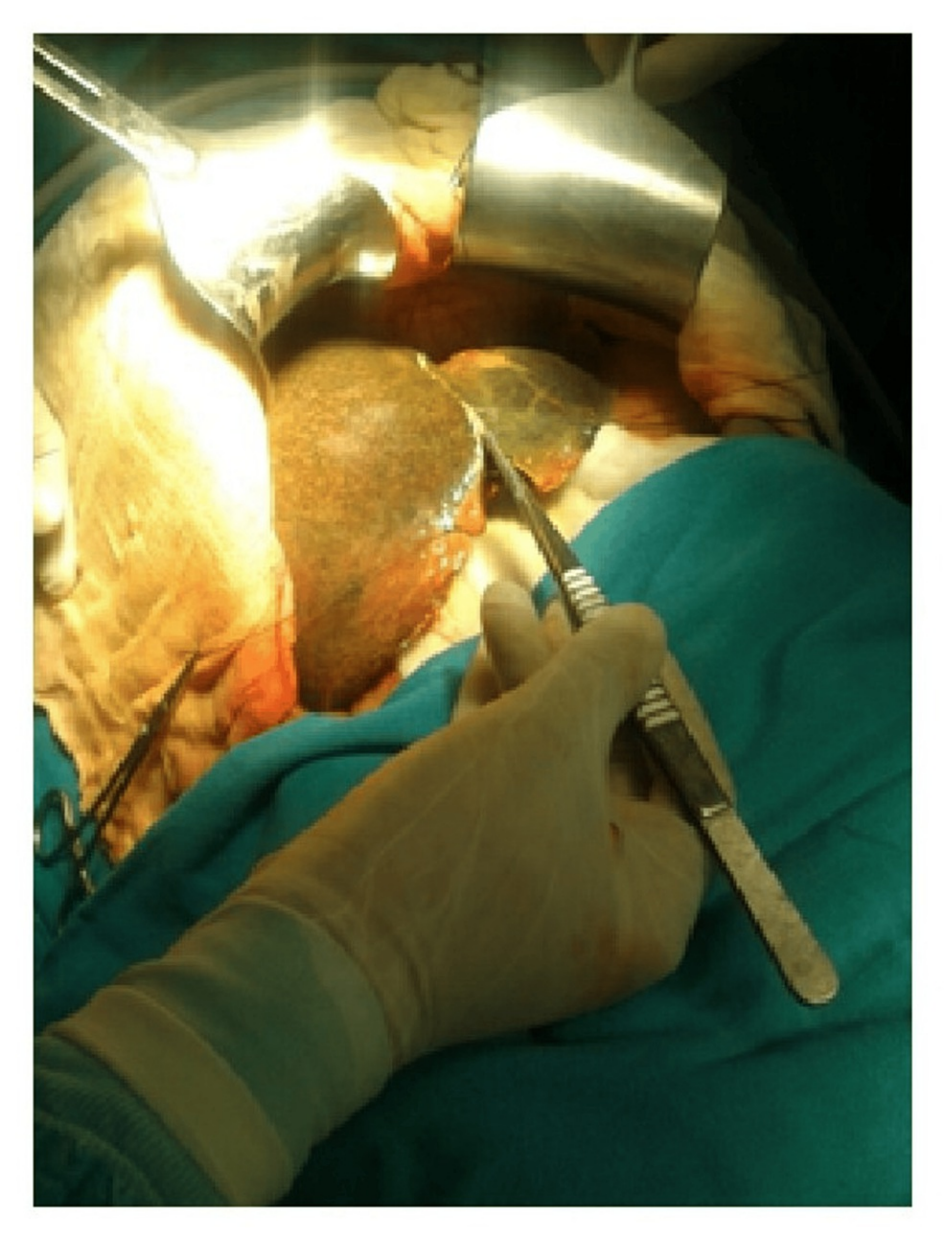
Neutral posture of the wrist while using DeBakey forceps. Image credit: [6].

**Figure 8:**
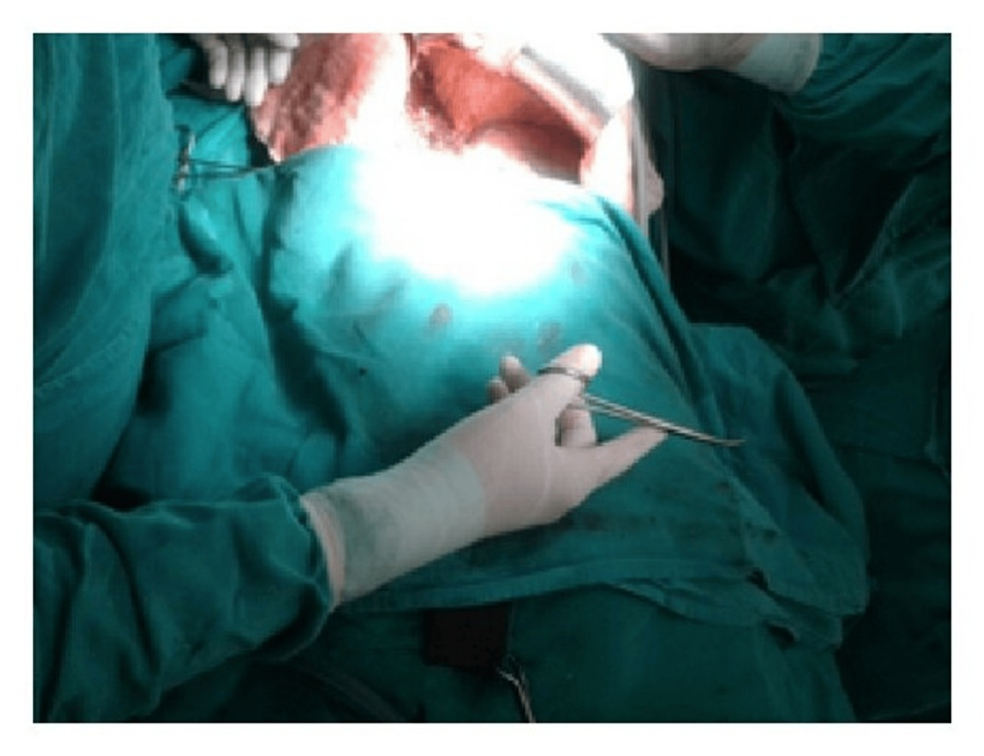
Position of the Kelly clamp with a neutral posture of the wrist. Image credit: [6].

**Figure 9:**
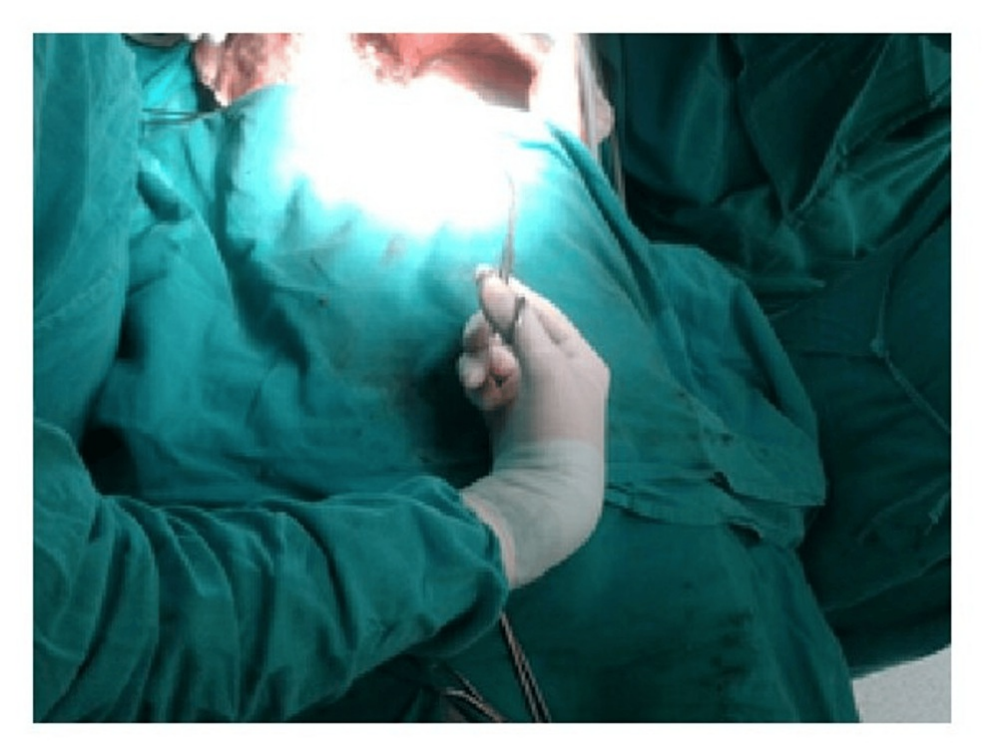
Functional position of Kelly clamp with an awkward posture of the wrist. Image credit: [6].

**Figure 10:**
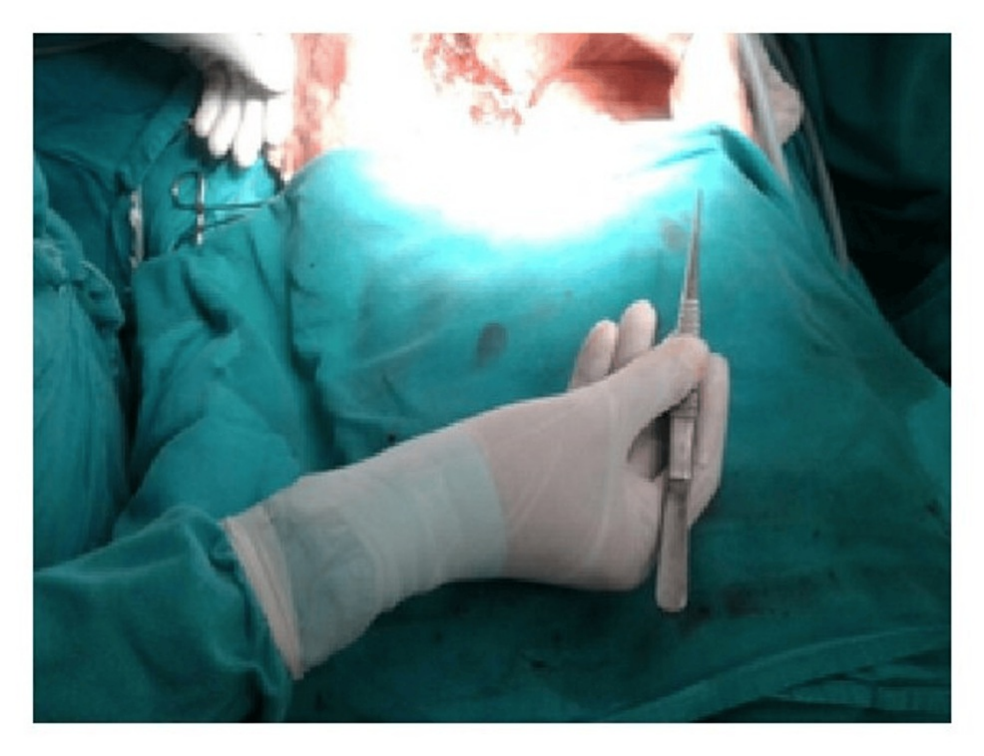
Position of DeBakey forceps with a neutral posture of the wrist. Image credit: [6].

Moreover, ergonomic instruments facilitate precise control and maneuverability, enabling surgeons to navigate complex anatomical structures more easily and accurately. This precision is crucial, particularly in delicate procedures involving intricate tissues or fine suturing, where even minor errors can have significant consequences for patient outcomes (Figures 6-10) [7].

**Figure 11:**
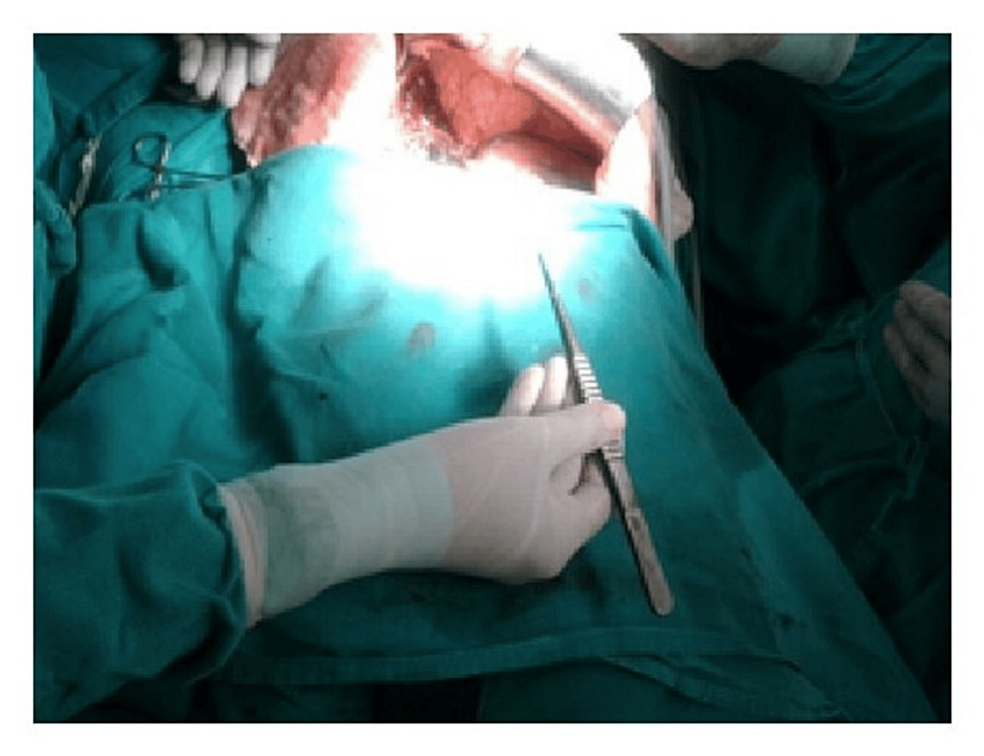
Inward position of DeBakey forceps with a neutral posture of the wrist. Image credit: [6].

**Figure 12:**
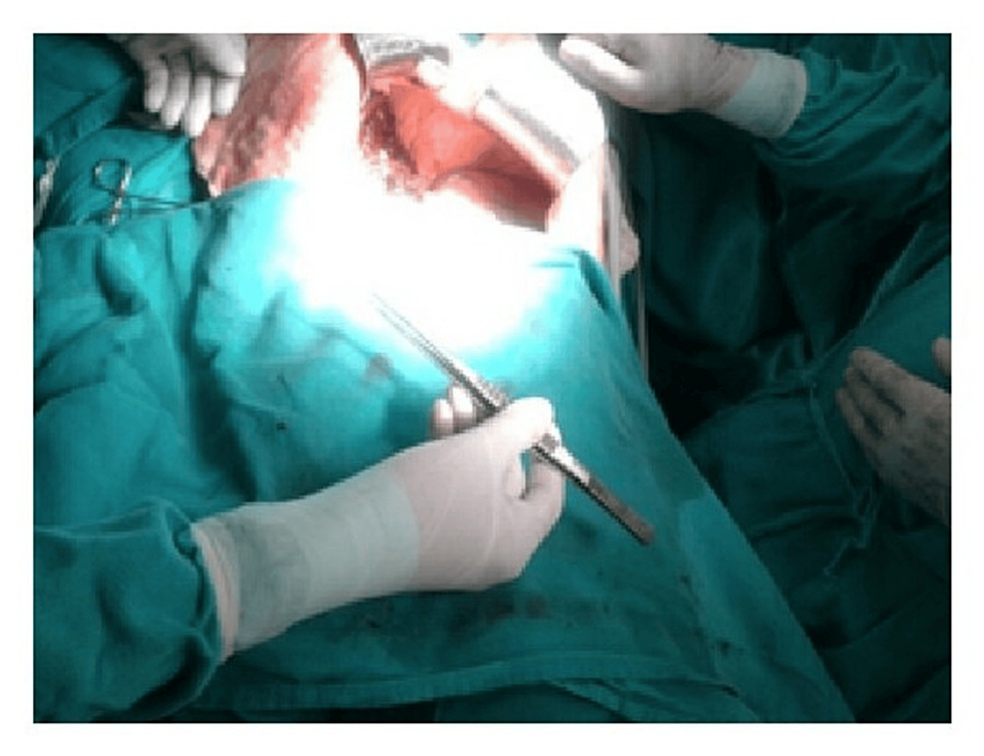
More inward position of DeBakey forceps with a neutral posture of the wrist. Image credit: [6].

Furthermore, ergonomic design promotes standardized grip techniques, ensuring consistency across surgical teams and reducing the risk of errors or mishaps during instrument handling. By providing intuitive feedback and tactile cues, ergonomic instruments empower surgeons to execute precise movements confidently, ultimately improving surgical outcomes and patient safety (Figures 11-12).

In essence, prioritizing the ergonomic aspect of surgical instruments enhances the surgeon's comfort and performance. It contributes to optimizing procedural efficiency, minimizing the risk of errors, and ultimately advancing the quality of patient care.

DeBakey forceps offer surgeons a natural hand posture, minimizing tissue damage during surgery and preventing fatigue while ensuring wrist stability. This ergonomic design enhances the surgeon's comfort and contributes to maintaining precision and control throughout the procedure [6,7,10,14,15].

By aligning with the hand's natural posture, DeBakey forceps reduce the strain on the surgeon's muscles and joints, promoting greater endurance during lengthy surgical interventions. This ergonomic advantage is particularly crucial in procedures requiring meticulous tissue manipulation, where even slight deviations in hand position can impact surgical outcomes [6].

Furthermore, the ergonomic design of DeBakey forceps helps mitigate tissue trauma risk by facilitating gentle yet secure grasping, preserving the integrity of delicate structures. This enhances patient safety and streamlines the surgical process by minimizing the need for corrective maneuvers or revisions.

The ergonomic features of DeBakey forceps prevent fatigue and promote precision, contributing to overall efficiency in the operating room. Surgeons can maintain optimal performance for extended periods, reducing the likelihood of errors and complications while promoting smoother surgical workflows.

The ergonomic benefits of DeBakey forceps extend beyond simply enhancing surgeon comfort. They play a crucial role in optimizing surgical outcomes, minimizing tissue trauma, and promoting procedural efficiency. By prioritizing ergonomic design, these instruments empower surgeons to deliver safer, more effective patient care.

Legacy of Dr. Michael DeBakey and the forceps bearing his name

Dr. Michael DeBakey's legacy extends beyond the development of forceps. He was instrumental in advancing cardiac surgery through his work on coronary artery bypass grafting, heart transplantation, and artificial heart development. He also played a significant role in medical education, training thousands of surgeons and medical professionals throughout his career.

DeBakey forceps stand as a testament to his innovative spirit and commitment to improving patient care. They continue to be used in operating rooms worldwide, symbolizing the lasting impact one individual's contributions can have on medicine [7].

## Conclusions

DeBakey forceps have had a transformative effect on cardiac surgery, enabling surgeons to perform complex procedures with greater precision and reduced risk of tissue damage. Their design and versatility have made them an indispensable tool in the surgical landscape, contributing to improved outcomes and the overall advancement of cardiac surgery. As cardiac surgery continues to evolve, the legacy of Dr. Michael DeBakey and the forceps that bear his name will remain integral to the ongoing pursuit of surgical excellence.
